# Age-specific growth and maturity estimates for the flatback sea turtle (*Natator depressus*) by skeletochronology

**DOI:** 10.1371/journal.pone.0271048

**Published:** 2022-07-20

**Authors:** Calandra N. Turner Tomaszewicz, Larisa Avens, Jeffrey A. Seminoff, Colin J. Limpus, Nancy N. FitzSimmons, Michael L. Guinea, Kellie L. Pendoley, Paul A. Whittock, Anna Vitenbergs, Scott D. Whiting, Anton D. Tucker

**Affiliations:** 1 NOAA Southwest Fisheries Science Center, La Jolla, CA, United States of America; 2 The Ocean Foundation, Washington, D.C., United States of America; 3 NOAA Southeast Fisheries Science Center, Beaufort, NC, United States of America; 4 Department of Environment and Science, Brisbane, QLD, Australia; 5 Charles Darwin University, Casuarina, NT, Australia; 6 Pendoley Environmental, Booragoon, WA, Australia; 7 Western Australia Department of Biodiversity, Conservation and Attractions, Perth, WA, Australia; Ocean Frontier Institute, CANADA

## Abstract

To address a major knowledge gap for flatback sea turtles (*Natator depressus*), a species endemic to Australia and considered ‘Data Deficient’ for IUCN Red List assessment, we present the first-ever skeletochronology-derived age and growth rate estimates for this species. Using a rare collection of bone samples gathered from across northern Australia, we applied skeletochronology and characterized the length-at-age relationship, established baseline growth rates from the hatchling to adult life stages, and produced empirical estimates of age-at- and size-at-sexual-maturation (ASM, SSM). We analyzed humeri from 74 flatback sea turtles ranging in body size from 6.0–96.0 cm curved carapace length (CCL), and recovered from Western Australia (n = 48), Eastern Australia (n = 13), central Australia (n = 8; Northern Territory n = 3, the Gulf of Carpentaria n = 5), and unknown locations (n = 5). We identified the onset of sexual maturity for 29 turtles, based on rapprochement growth patterns in the bones. Estimates for ASM ranged from 12.0 to 23.0 years (mean: 16.3 ± 0.53 SE), SSM ranged from 76.1 to 94.0 cm CCL (mean: 84.9 ± 0.90 SE), and maximum observed reproductive longevity was 31 years for a 45-year old male flatback. Growth was modeled as a smoothing spline fit to the size-at-age relationship and at the mean SSM (84.9 cm CCL) corresponded with a spline-predicted maturity age of 18 years (95% CI: 16 to 24), while mean nesting sizes reported in the literature (86.4 to 94 cm CCL) corresponded to estimated ages of 24+ years. A bootstrapped von Bertalanffy growth model was also applied and showed consistencies with the spline curve, yielding an estimated upper size limit, *L*_*inf*_, at 89.2 ± 0.04 cm (95% CI: 85.5 to 95.9 cm) with the intrinsic growth rate parameter, *k*, at 0.185 ± 0.0004 (0.16 to 0.22); at the same mean SSM (84.9 cm CCL) the estimated ASM was 16.3 ± 0.05 years (95% CI: 12.8 to 27.7 years). Lastly, four of the samples analyzed were collected from deceased adult females that had previous sizes known from on-going mark/recapture studies at nesting sites in Western Australia. The paired CCL data (measured at nesting and back-calculated) did not significantly differ (p = 0.875). This first skeletochronology study for flatback sea turtles generates valuable empirical estimates for ongoing conservation and management efforts.

## Introduction

Effective conservation and management of threatened and endangered species is ideally founded upon understanding basic life history parameters including age-to-maturity, reproductive longevity, life span, and age-specific growth rates [1–3]. Threatened species assessments (e.g., the International Union for the Conservation of Nature, IUCN, Red List), population-level recovery plans, and management actions should rely on the input of robust quantitative biological data which is best collected from *in situ* samples and encounters with wild animals [4–6]. Yet for many marine species, especially those that are long-lived, slow to mature, and occupy distant and remote habitats, estimates of some key demographic parameters can be difficult to obtain [3, 7]. Sea turtles are one taxon for which life history parameters and vital rates have been challenging to estimate [3, 8]. While numerous studies exist documenting growth rates obtained from longitudinal mark-recapture efforts [3, 9, review in 10], many of these studies are limited to only the juvenile or adult life stages depending on which age classes are present at capture locations, and do not yield age-specific information over the lifetime of the animals. For instance, *in situ* observations of age-at-first nesting and reproductive longevity have been documented from a handful of sea turtle populations through long-term capture-mark-recapture (CMR) projects at nesting beaches and foraging areas (e.g., flatback sea turtles, *Natator depressus* [11, 12]; green sea turtles, *Chelonia mydas* [13]; loggerhead sea turtles, *Caretta caretta*, summarized in [14]; Kemps ridley, *Lepidochelys kempii* [15]). The tagging of hatchlings and head-started post-hatchlings has also contributed to knowledge on age at first breeding and other important demographic information (e.g., [11, 12, 16, 17]). At the molecular level, advances in genetic fingerprinting during saturation tagging efforts at nesting beaches are nearing age-at-first-reproduction results (e.g., leatherback sea turtles, *Dermochelys coriacea* [18]), and new genomic techniques which quantify specific protein densities have yielded initial estimates of lifespan for several sea turtle species [19, 20]. Such mark-recapture and genetic tagging studies are extremely valuable and informative for providing initial parameter estimates for maturation age and longevity, but they remain limited in the number of individual sea turtles aged and in the scope of species and populations assessed.

A robust approach to estimate critical age-specific demographic rates and population-level variation of these parameters is to use skeletochronology, the study of growth layers of bones [9, 21, 22]. Since the mid-1980’s, numerous studies have characterized the relationships of size, age, and growth of sea turtles, including the timing of maturity, by analyzing the bones (humerus or scleral ossicle) from dead turtles found stranded or as bycatch [9, 21, 23]. Of the seven extant sea turtle species, the flatback sea turtle remains distinct as having never been analyzed using skeletochronology (reviewed in [9]), with all other sea turtle species, green, loggerhead, hawksbill, olive ridley *Lepidochelys olivacea*, Kemp’s ridley and leatherback, having been studied for age and growth rates using bones (reviewed in [9, 23]). One of the reasons why Australian flatbacks have yet to be analyzed for age and growth by skeletochronology was the difficulty and lack of priority in collecting samples. As a result, a knowledge gap exists regarding the age-specific life history and growth rate parameter estimates for flatback sea turtles, a species listed as “Vulnerable” in the Australian (Commonwealth) Environmental Protection and Biodiversity Conservation Act [24]. These unknowns—together with the absence of long-term abundance data, mortality data, and foraging ground habitat use data—are all reasons why the species is categorized as Data Deficient in species (and stock) status reports such as the IUCN Red List [25].

### Australian flatback sea turtles

The flatback sea turtle differs from the other five species of hard-shelled sea turtles in the *Cheloniidae* family for multiple reasons. Flatbacks nest only in Australia, have eggs and hatchlings that are much larger proportional to adult body size than any other sea turtle species, do not have an oceanic juvenile stage, have superior breath-holding capacity compared to other Cheloniid turtles, and have a thin skin-like layer of keratinous carapace scutes [26–29]. This species is almost totally restricted in distribution to a single continental region: nesting along northern Australian beaches and foraging in continental shelf waters of northern Australia, and southern New Guinea in Indonesia and Papua New Guinea [27]. Genetic analysis of nesting flatbacks have revealed at least seven distinct genetic stocks (or breeding groups), with some groups potentially further segregated by nesting season (winter vs. summer) [30]. Genetic differentiation, in both mitochondrial and nuclear DNA, even among rookeries within relatively close proximity to one another (i.e., < 300km apart [30, 31]), suggests strong natal homing for both males and females, despite geographically large foraging home ranges and migrations of up to 1500km [32, 33].

The large size of flatback hatchlings (straight and curved carapace length ~6 cm [27]) is thought to provide an increased survival rate of hatchlings [34] and greater diving capabilities for post-hatchling flatbacks that disperse into and successfully forage within neritic shelf waters after hatching [26, 35, 36]. Unlike other sea turtle species, flatbacks do not have an oceanic juvenile stage, instead remaining in the continental shelf waters of Australia, consuming pelagic and benthic invertebrates [27, 29, 32]. Flatbacks of less than 30 cm curved carapace length (CCL) have been found dead-stranded at widely dispersed sites across the northeastern Australian beaches, in stomachs of various fish and shark species, surface set gill nets, as well as at island feeding stations of white-bellied sea-eagles (*Haliaeetus leucogaster*) in the Great Barrier Reef, supporting the theory that juveniles remain in neritic habitats throughout development [26, 27, 37]. While some flatbacks in the 50–70 cm CCL size range have been encountered during recent surveys at foraging grounds in northern Australia at Roebuck Bay (DBCA unpub.), strikingly few flatback sea turtles have ever been observed in this size range by fisheries trawl sampling, making it difficult to estimate growth rates during the juvenile life stages of wild (non-captive) flatbacks. In contrast, during transects targeting convergence lines in Keppel Bay in central eastern Queensland, post-hatchling flatback sea turtles (and no other species of sea turtles) of less than 30 cm CCL are regularly observed/captured while feeding on macro zooplankton at the surface within 15–20 km of the shore (DES Queensland Turtle Conservation Database, unpubl.).

Adult female flatbacks from all breeding groups typically begin nesting between 75 and 95 cm CCL (reviewed in [27]). Robust information on age and growth rate parameters of adult flatbacks comes from the Queensland Turtle Project program (DES Queensland Turtle Conservation Project [11]). Efforts starting in 1974 to mark thousands of hatchlings and recapture them as nesting adults found one tagged turtle so far which returned to nest at age 21 [12, 27], giving one confirmed age-at-sexual-maturation (ASM) record. The introduction of titanium tag use in 1981 and subsequent use of PIT tags in Queensland facilitated long-term identification of individual sea turtles at the nesting beach in the wild [38] with long-term monitoring documenting numerous sea turtles nesting for in excess of 35 years at multiple rookeries and currently with a maximum value of 47+ years (in [27], DES Queensland Turtle Conservation Project, unpub.). When summed, these values (ASM + reproductive longevity) suggest a total lifespan of greater than 68 years for flatback sea turtles. Mayne et al. [19] applied a new genetic technique (epigenetics) and found a correlation of lifespan of vertebrates with that of the densities of specific DNA methylation regions, specifically at cytosine-guanine, CpG, sites in gene promoters. Using this genomic approach, Mayne et al. [20] estimated the lifespan of six sea turtle species, including flatbacks, with flatbacks having the shortest estimated lifespan at 50.4 ± 2.9 years, a possible underestimate when considering the estimates collected *in situ* for wild tagged (mark-recaptured) flatback sea turtles ([27], DES Queensland Turtle Conservation Project, unpub.). These early findings provide needed demographic parameters for population recovery plans and stock status assessments. However, the small sample size from the *in situ* studies and the fact that the genomic approaches are still being tested and validated, leave major knowledge gaps about the life history and demography of flatback sea turtles.

In the current study, we applied skeletochronology techniques to humerus samples from museums and permitted collections in Queensland, Northern Territory, and Western Australia, augmented by field efforts during 2013–2021 to address this knowledge-gap for flatbacks. The specific aims of this study were to characterize the length-at-age relationship, establish baseline values for growth rates from the hatchling to adult life stages, and produce empirical estimates of age-at- and size-at-sexual-maturation for the flatback species as a whole.

## Methods

### Sample collection and processing

**Fig 1:**
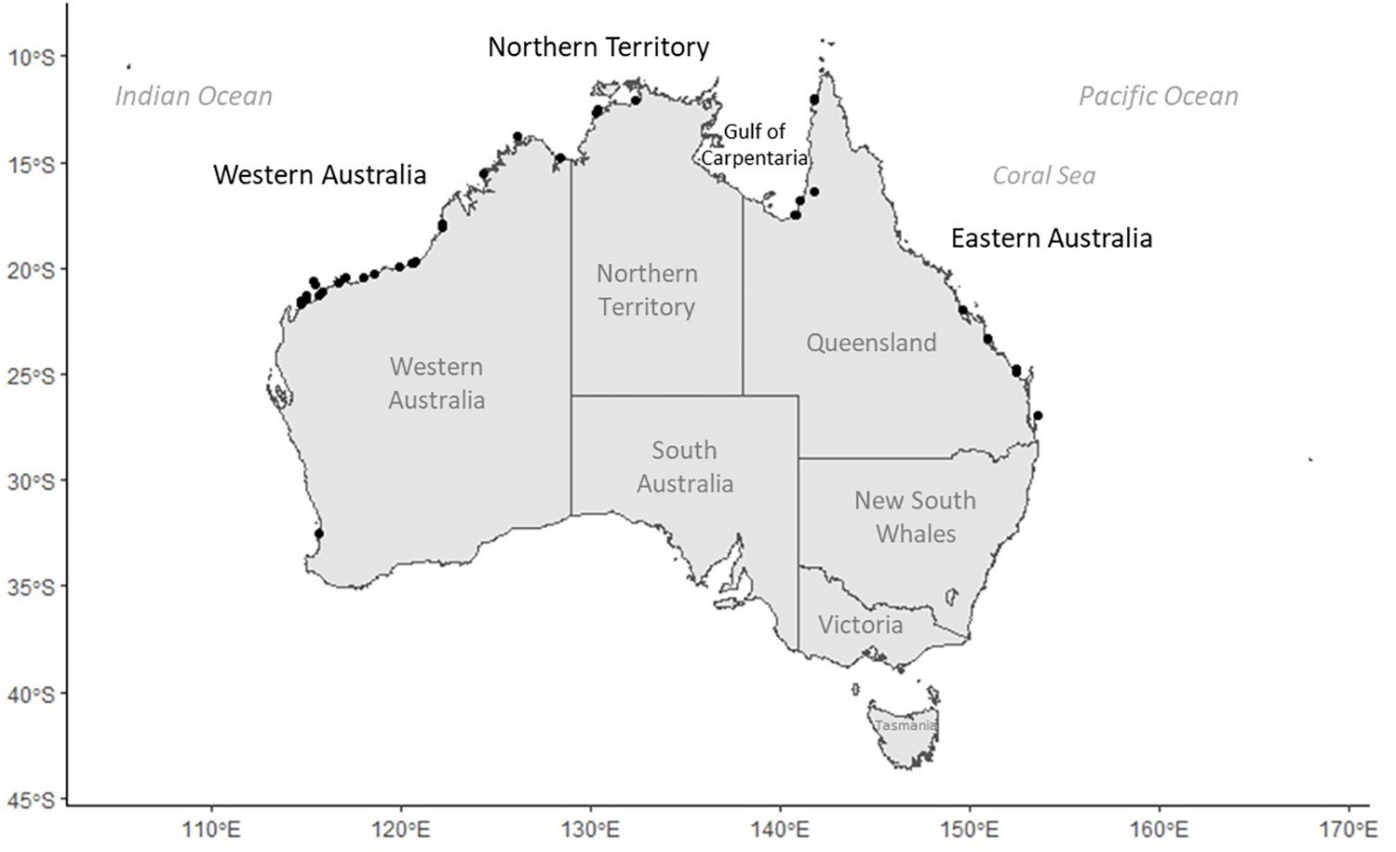
Study area. Map showing location of samples collected from 4 regions: Western Australia, Northern Territory, Gulf of Carpentaria, and Eastern Australia. (Map made using open source R software and R package ‘ggplot2’ (https://github.com/tidyverse/ggplot2) and ‘ozmaps’ (https://github.com/mdsumner/ozmaps).

We used humeri collected from dead flatback sea turtles that stranded along the northern Australian coast (Fig 1). All samples for this study were collected from already-deceased animals, with full permits for collection and CITES transport. Upon recovery of stranded carcasses, turtles were searched for flipper and passive integrated transponder (PIT) tags, body size was measured as curved carapace length, CCL (aka, “ccl min”, herein referred to as CCL: from the nuchal notch along the midline to between the post marginal scutes [39]). Bone samples were collected by dissecting out the humerus in the field, boiled to remove excess flesh, and air dried until processing. When possible, sex was inferred based on nesting history or gonadal examination during necropsy.

Dried humeri were cut and processed for skeletochronology analysis as fully described in [40]. Briefly, we cross-sectioned two 3-mm pieces from each humerus using an Isomet slow speed saw and diamond wafer blade. One section was used in the current study, and was fixed in 10% buffered formalin and decalcified in a commercial “RDO” solution by Apex, before being sliced into 25-μm-thin sections using a freezing-stage sliding microtome by Leica. Thin sections were then stained in a modified Ehrlich’s stain, visually inspected for completeness, mounted to a slide, and then digitally imaged to produce a high-resolution record of the bone revealing lines of arrested growth (LAGs) that denote the outer edges of individual skeletal growth marks. Imaging was conducted on an Olympus CX43 microscope using cellSens software. In hard-shelled sea turtles, LAGs have been shown to form annually in multiple species (loggerhead, Kemp’s ridley [41], green [42, 43], hawksbill [44]), and this was assumed true in the current study, with formal validation of yearly LAG formation in flatbacks encouraged in future studies.

Digitized images were then visually inspected by at least two independent readers (CTT, LA, LRG), following standard skeletochronology protocol, and each person marked the location and total count of LAGs observed in each bone [9, 45, 46]. The marked reads were compiled for a final consensus to determine the LAG location and number for all bones. Recorded LAG diameters were then measured along the antero-posterior axis [21], and the data were used for age, size and growth estimation analyses as described below [47].

### Age, size and growth

To estimate age, somatic growth, and body size at each LAG, we applied standard skeletochronology analysis (see [9, 48, 49]). We first evaluated the relationship between total humerus section diameter (THD) and turtle body size CCL. If there was a strong relationship (r^2^ > 0.9) between THD and CCL, then minimum and maximum LAG diameter measurements for each bone were used to group samples for age and size estimation. Estimated age was generated by summing the number of LAGs observed in a bone, with the number of estimated LAGs lost interior to each bone’s minimum-diameter LAG or resorption core. Interior LAGs in sea turtle bones are lost or underestimated due to resorption that occurs during bone growth, and for turtles with bones exhibiting resorption, the number of LAGs lost was estimated by using the standard skeletochronology correction factor (CF) approach [9, 48, 50].

The CF approach is based on developing a stepwise relationship between the LAG diameter and corresponding LAG numbers from the bones of smaller flatback sea turtles, starting with those turtles that retain the year-1 annulus (the LAG formed during the first year of a sea turtle’s life), to then estimate the number of missing LAGs from the bones of larger flatback sea turtles, given the size of each bone’s resorption core. Here, we used parametric and non-parametric methods to model this relationship between LAG number and LAG diameter for humeri containing an annulus, and selected the best model based on r^2^ values [50], to estimate the number of LAGs lost due to resorption in the larger turtle bones. To ensure consistency among bones, we used the diameter of the innermost measurable LAG of each larger turtle bone (those missing its annulus) as a proxy for resorption core diameter during further analysis.

Lastly, because 1) flatback bones are exceedingly rare samples opportunistically collected from across the entire northern Australian coast with inherent uneven geographic distribution, and 2) flatbacks in this wide area are known to nest in the summer (e.g., Mundabullangana) and the winter (e.g., Bare Sand Island and Cape Domett, [27]), we did not attempt to distinguish the timing of LAG formation nor a single peaking hatching month for individual bones, as sometimes done in sea turtle skeletochronology studies with larger sample sizes from a single or known nesting region. As a result, we did not assign partial-year growth or age. Instead, we took a conservative approach to best estimate baseline vital rates for flatbacks by analyzing all samples together, regardless of stranding location, season, year or genetically distinct breeding group [30], and rounded all estimated ages to the nearest whole number.

Once the final age was estimated for each bone, we assigned an estimated age to each subsequent LAG by subtracting one for each LAG moving inward from the outermost LAG. Next, for each bone, we back-calculated body size (CCL) at each measurable LAG to produce estimated size as well as incremental growth, as fully described in [47, 48, 51]. Using this approach, we applied the body proportional hypothesis (BPH)-corrected [52] allometric equation modified for application to sea turtles to yield a back-calculated CCL for each turtle at each measured LAG as recommended in Snover et al. (2007) and commonly applied in other sea turtle studies [9, 49, 51]. To do this we first characterized the relationship between CCL and humerus section diameter using the following allometric equation [47, 53]:

$$L=L_{op}+b{\left(D-D_{op}\right)}^{c}$$ [Eq 1]

Here, *L* is the estimated length (CCL), *L*_*op*_ is the minimum hatchling CCL, *D* is the humerus section diameter, *D*_*op*_ is the minimum hatchling humerus diameter, *b* is the slope of the relationship, and *c* is the proportional coefficient. To obtain values for flatback hatchling CCL and humerus diameter we collected and measured carapace length and humeri diameter of 66 hatchlings that had been collected and used in separate on-going studies, and recorded minimum humerus diameter (2.4 mm) and minimum CCL (6 cm). Parameters *b* and *c* were optimized using the nonlinear least squares function ‘nls’ in the ‘stats’ package in R version 4.0.1 [54]. Next, to apply this relationship to LAG diameters, we adjusted Eq 1 such that LAG diameter was used in place of humerus section diameter, to yield a back-calculated BPH-corrected body size (CCL) at time of LAG formation [47]:

$$L_{initial}=[L_{op}+b{\left(D_{initial}-D_{op}\right)}^{c}]\times [L_{final}]\times {\left[L_{op}+b{\left(D_{final}-D_{op}\right)}^{c}\right]}^{-1}$$ [Eq 2]

For any turtles with a nesting history and recorded CCL, the CCL for that year and LAG-back-calculated CCL were compared with a paired-sample Wilcoxon rank sum test. For any turtles observed nesting more than one year (i.e. CCLs recorded from multiple years), the mean value of all paired samples was used for each individual turtle for the Wilcoxon rank sum test to eliminate any pseudoreplication from non-independent repeated measures.

We then used these back-calculated CCLs and analyzed annual somatic growth rates of individual turtles, as well as size and age class groups by calculating the incremental growth between adjacent LAGs, as done in other skeletochronology studies (i.e. [47, 48]). For turtles that retained an annulus (year-1 mark), somatic growth during year-1 was calculated by subtracting the minimum flatback hatchling size (6 cm, current study) from the back-calculated CCL corresponding with each turtle’s annulus. Taken together, we created a multi-year record of size, age, and growth for each individual turtle by grouping the corresponding results for each turtle’s LAG.

To characterize the size-at-age relationship for all turtles together, we used a generalized additive mixed model (GAMM) fitted with a cubic smoothing spline and modeled growth using a bootstrapped Fabens-modified von Bertalanffy growth model (VBGM), similar to the process conducted in [55, 56]. This analysis, applying and comparing both a parametric (Fabens-modified VBGM) and a nonparametric (GAMM) model, allowed us to compare both approaches, especially to evaluate potential convergence when approaching sizes near maturation. Application of the VBGM was also useful because it also allowed for direct comparison with other prior studies reporting results of parametric growth models [23, 53, 57, 58]. The GAMM accounted for individual turtle size and growth variation and repeated observations (mixed longitudinal sampling design) by incorporating a turtle-specific random effect, as commonly applied and fully described in other skeletochronology and growth analysis studies (e.g., [48, 49, 59–61]). Analysis was conducted using the ‘mgcv’ package and the ‘gamm’ function in R version 4.0.1 [54, 62]. We then fit a smoothing spline model to the size-at-age data back-calculated through conversion of all measurable LAG diameters to estimates of CCL, to characterize the estimated size-at-age data and the predicted fit with 95% confidence intervals (CI) using the ‘smooth.spline’ function in the ‘stats’ package in R [54, 63]. Next, we modeled growth using a bootstrapped method of the VBGM, applied and fully described in [48, 55], where the back-calculated somatic growth rate data were repeatedly sampled to extract a single growth-at-length data point for each individual turtle in the sample. These nonparametric bootstrap samples were then used to estimate the growth parameter, *k*, and estimated upper size limit, *L*_*inf*_, for Fabens modification of the von Bertalanffy growth curve. For this VBGM, randomized re-sampling of the growth rate data was conducted 1,000 times to describe uncertainty in the von Bertalanffy parameters [48, 55, 56]. We examined age-at-sexual-maturation (ASM) variation from different geographic regions by using both the smoothing spline and von Bertalanffy growth curves to yield ASM estimates as the age corresponding with different size-at-sexual-maturation (SSM) estimates from the literature, and we compared the mean nesting sizes as well as the minimum sizes reported for the different geographic regions represented by samples used in the current study (Western Australia, Northern Territory, Gulf of Carpentaria, and Eastern Australia).

For the adult turtles, we also estimated the onset of maturation (ASM and SSM) and minimum reproductive longevity, based on the location of the bone where we observed compression of the LAGs at the lateral edge of the bone, termed rapprochement [9, 48, 64]. Rapprochement in the bones occurs when somatic growth slows upon reaching maturity, when resources are shifted away from body growth and into reproduction [9, 64]. We quantitatively assigned this maturation point as the first LAG where at least three sequential growth increment estimates were ≤ 0.5 cm, as this is the standard protocol for skeletochronology analysis [48]. We calculated minimum reproductive longevity as the total number of LAGs observed from the maturation-LAG and moving outward to the bone edge. Post-maturation LAGs, and corresponding age, year, and CCL, were also compared with nesting history observations of tagged turtles when possible, as a way to begin validating assumptions used in skeletochronology (e.g., annual LAG formation, CCL back-calculation) for flatback sea turtles. For all analyses, significance was evaluated as p < 0.05, and results are presented as mean ± standard error (SE) unless otherwise noted.

## Results

### Age and growth estimation

A total of 74 flatback humeri were analyzed: 27 were hatchlings/post-hatchlings (only retained hatch-mark, CCL: range 6.0 to 17.0 cm, mean ± SE 9.4 ± 0.64 cm), 15 were neonatal to juvenile (CCL: 17.6 to 36.6 cm, mean ± SE 26.6 ± 1.5 cm), and 32 were putative adults (CCL: 78.8 to 96.0 cm, mean ± SE 87.5 ± 0.8 cm). Most of the sea turtles were recovered from Western Australia (n = 48) and the rest were from three other geographic regions: Eastern Australia (n = 13), the Northern Territory (n = 3) and the Gulf of Carpentaria, Queensland (n = 3), with five from unknown locations (Table 1, Fig 1). Of the 32 putative adults, sex was known for 17 of the turtles: 15 females known from flipper tags and/or nesting histories, and two males determined from gonadal examination at necropsies (data in S1 File). The CCL was recorded at the time of carcass recovery for 59 of the 73 turtles, and the body size of the 14 turtles without recorded CCL was estimated based on the following linear relationship between total humerus section diameter (THD) and turtle size (CCL):

CCL=2.845×THD+0.367, [Eq 3]

adj. r^2^ = 0.99, S1 Fig in S3 File

**Table 1:** Flatback sample details.

Location	n	Mean ± SE	Range
Eastern Australia	13	44.1 ± 8.7	11.3 to 96.0
Gulf of Carpentaria	5	27.8 ± 2.5	21.6 to 35.9
Northern Territory	3	83.5 ± 3.9	78.8 to 91.2
Western Australia	48	48.1 ± 5.7	6 to 94.3
Unknown	5	37.1 ± 18.2	7.2 to 83.0

Sample sizes and curved carapace length (CCL, cm) showing means, standard error (SE) and minimum and maximum by stranding location.

We identified and measured a total of 690 LAGs from among the 73 humeri that were able to be fully processed (one putative adult bone was too brittle), with individual turtles retaining between 0 to 42 (mean ± SE 9.3 ± 1.3) LAGs. The 27 hatchling/post-hatchling bones retained only a hatch mark, with no annual LAGs. A total of 15 humeri retained an annulus (the LAG formed during the first year of a sea turtle’s life) and these bones (Group 1) were directly aged following standard sea turtle skeletochronology-based aging protocols (see [40, 61]), where one LAG = 1 year. The remaining fully processed bones (n = 31, putative adults, Group 2) did not retain an annulus, meaning that at least one LAG had been lost (resorbed) during bone growth (data in S1 File).

**Fig 2:**
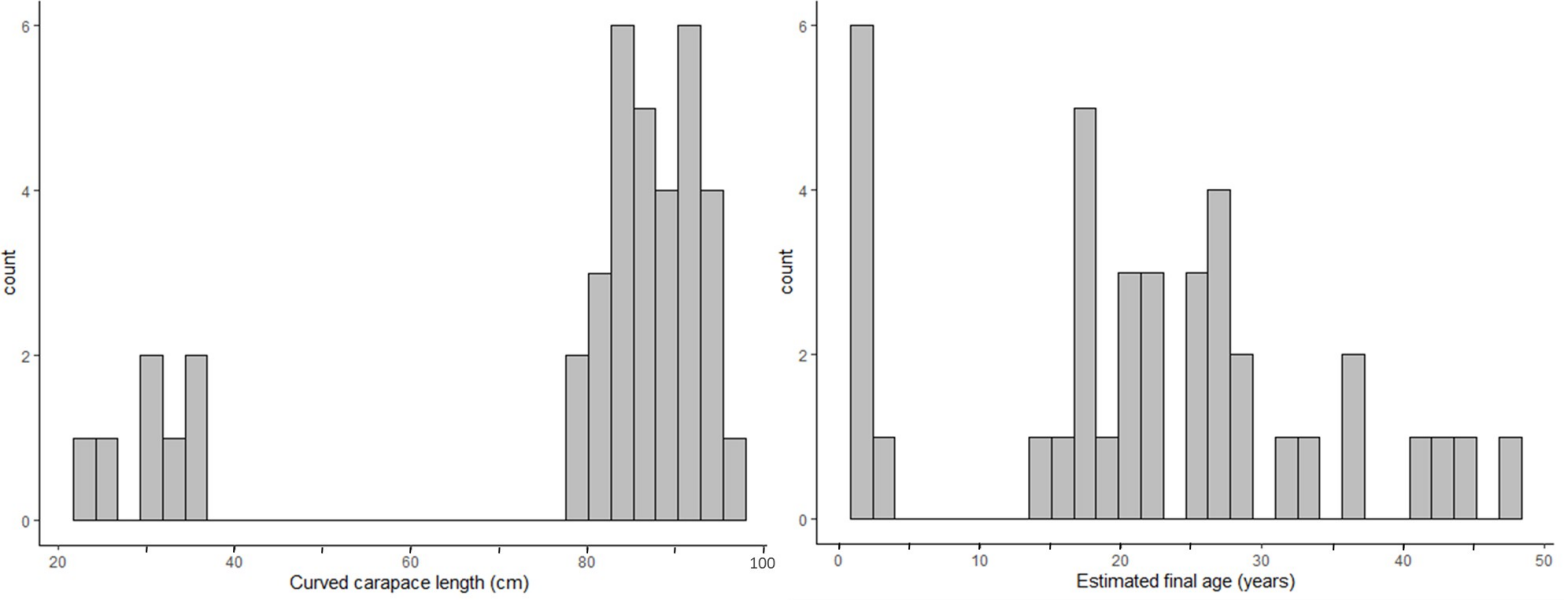
Size and age at stranding. Distribution of non-hatchling flatback sea turtles’ size and estimated age at stranding (n = 38).

For these Group 2 bones, we determined the best-fit correction factor equation (CF1) from the directly-aged Group 1 bones (n = 15 turtles, 23 LAGs, max LAG diameter or THD: 12.5 mm, S2 Fig in S3 File) which was a second order polynomial:

$$CF1:y=5.53+1.38x+0.227x^{2},\;r^{2}=0.584$$ [Eq 4]

where *y* is the LAG diameter, *x* is the LAG number. Using this CF1 equation, we estimated the number of LAGs lost due to resorption, where LAG diameter *y* was each bone’s innermost LAG or resorption core diameter. We then summed this resulting LAG number with the number of LAGs retained in each bone to obtain final estimated age at stranding (Fig 2, data in S2 File).

The number of estimated LAGs lost (resorbed) from the individual putative adult bones ranged from four to eight (mean ± SE 5.8 ± 0.19) LAGs. Final estimated age (LAGs retained + LAGs resorbed, with all ages rounded to the nearest whole number) for the 31 putative adults ranged from 15 to 48 years old, (mean ± SE 26.6 ± 1.63 years), and for all 46 non-hatchling turtles combined, estimated age ranged from one to 48 years old, (mean ± SE 18.4 ± 2.06 years (Fig 2).

**Fig 3:**
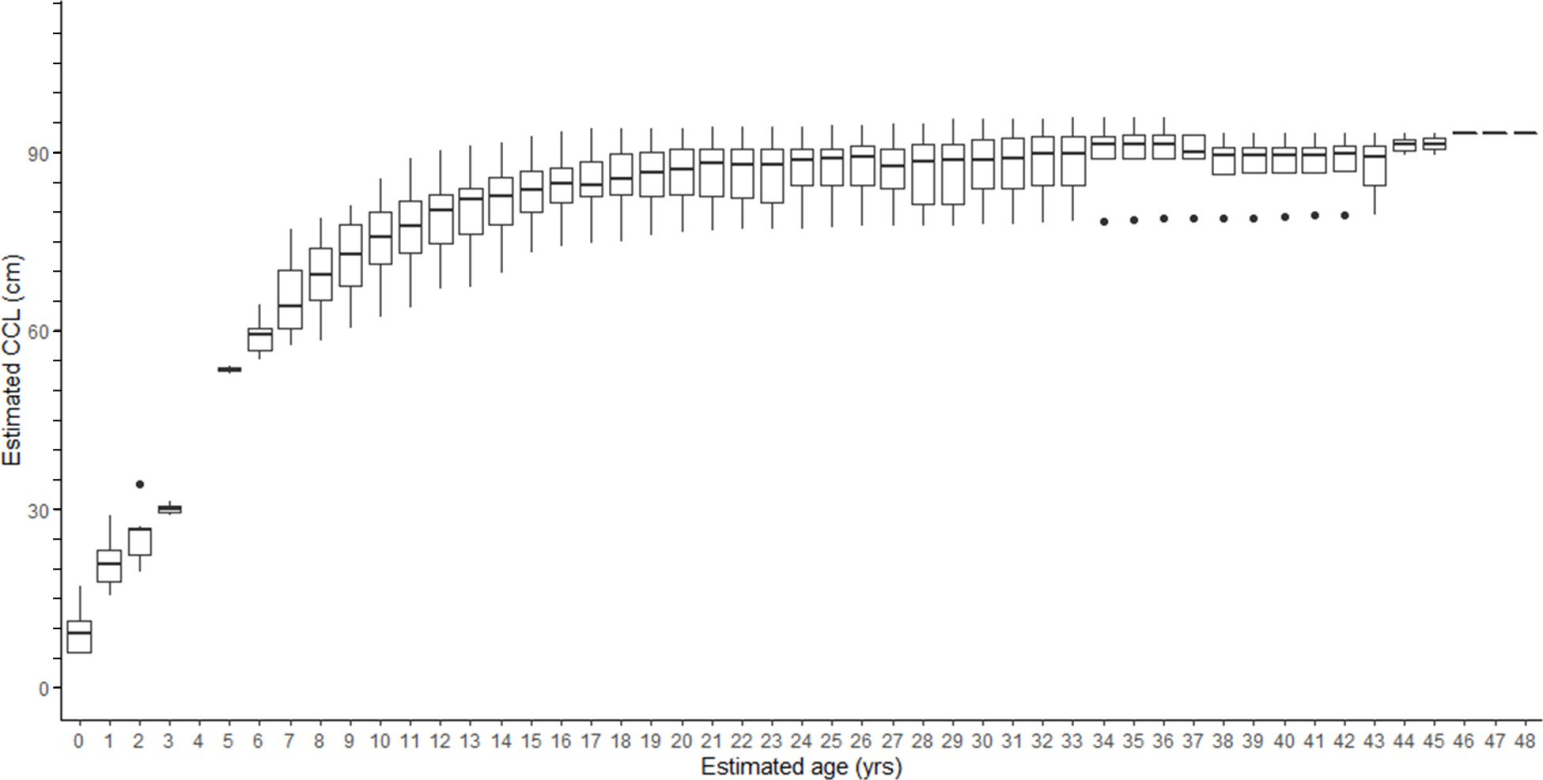
Flatback size-at-age. Range of estimated size (curved carapace length, CCL, cm) at each estimated age (years) for all 72 aged flatback sea turtles.

**Fig 4:**
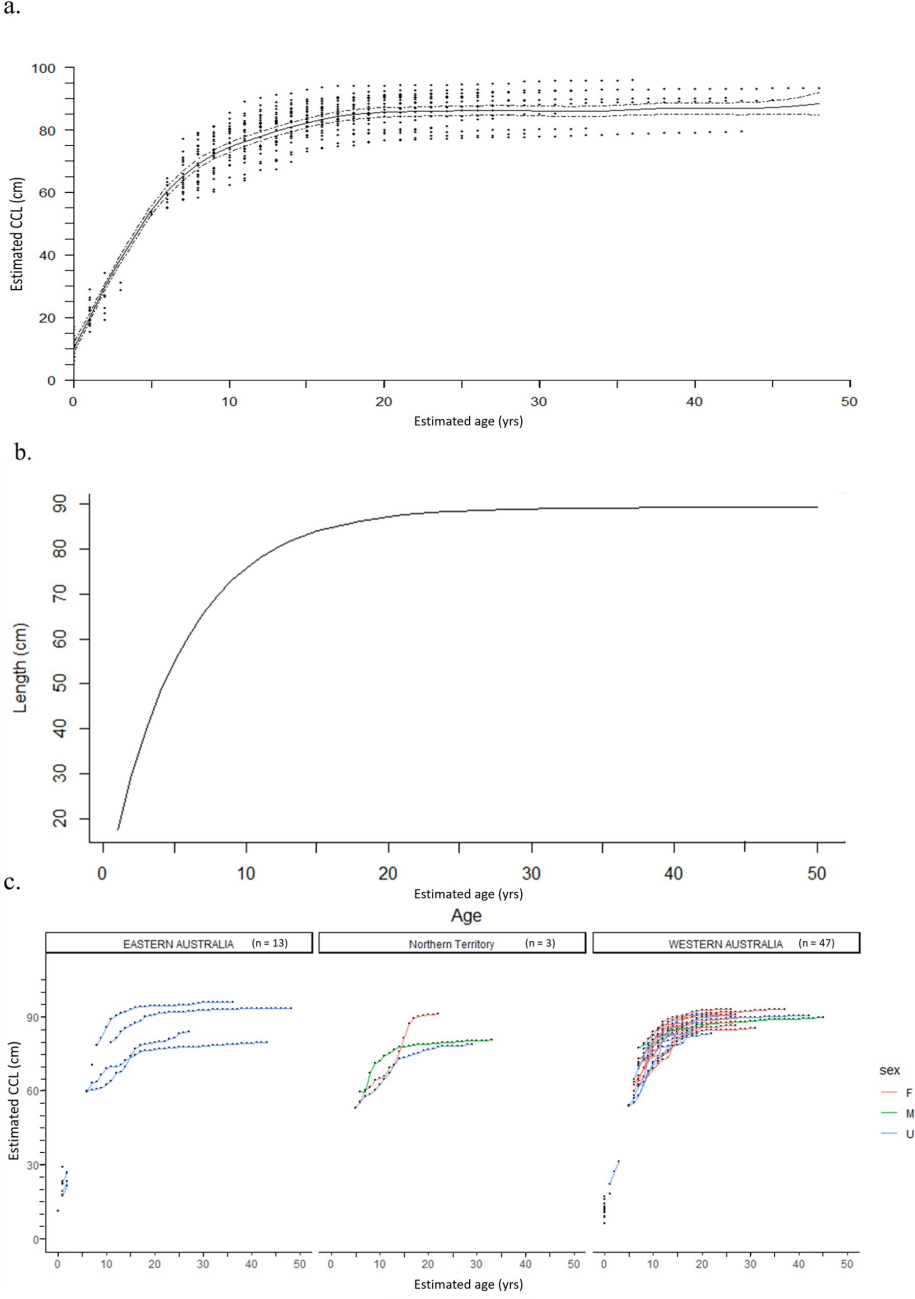
Growth curves. a) Back-calculated estimated curved carapace length (CCL) and corresponding estimated age (years) for 72 flatback sea turtles with the generalized additive mixed model (GAMM) smoothing spline (solid line) and 95% CI (dashed lines). b) von Bertalanffy growth curve using growth increments and estimated CCL. See *Methods* for details. c) size and age of turtles separated by geographic regions, Eastern Australia, Northern Territory, and Western Australia, to show samples collected at each location.

Typically, all of the Group 2 bones would each have a resorption core or minimum LAG diameter that is less than the maximum LAG diameter or THD of the directly-aged Group 1 bones (12.5 mm, S2 Fig in S3 File). However, there were zero flatback sea turtle carcasses recovered with CCL between 40 to 70 cm CCL, and no Group 2 humeri retained an innermost LAG or resorption core diameter that was less than the maximum THD from the Group 1 bones. In the current study, we therefore had to apply CF1 to all of the Group 2 bones, which had minimum LAG diameters ranging from 16.7 to 29.1 mm, knowing the resulting estimate of resorbed LAG number could be improved in future studies with additional samples from the missing intermediate size range. For all 73 bones, we applied the flatback-specific hatchling parameters (min CCL: 6 cm, min hatchling THD: 2.4 mm, slope *b* = 3.665, proportionality coefficient *c* = 0.928) to the BPH back-calculated CCL equation (Eq 2), at each measurable LAG which yielded 690 CCL-at-age estimates (Fig 3), and 629 annual somatic growth estimates (S3 Fig in S3 File). Annual CCL growth estimated from bone LAGs ranged from zero to 23.0 cm/yr. As expected, annual growth was greatest for age zero to one, mean 15.1 cm/yr, and decreased monotonically as turtles grew (Fig 4, S3 Fig, S1 Table in S3 File, data in S2 File).

### Age- and size- at-sexual-maturation

**Fig 5:**
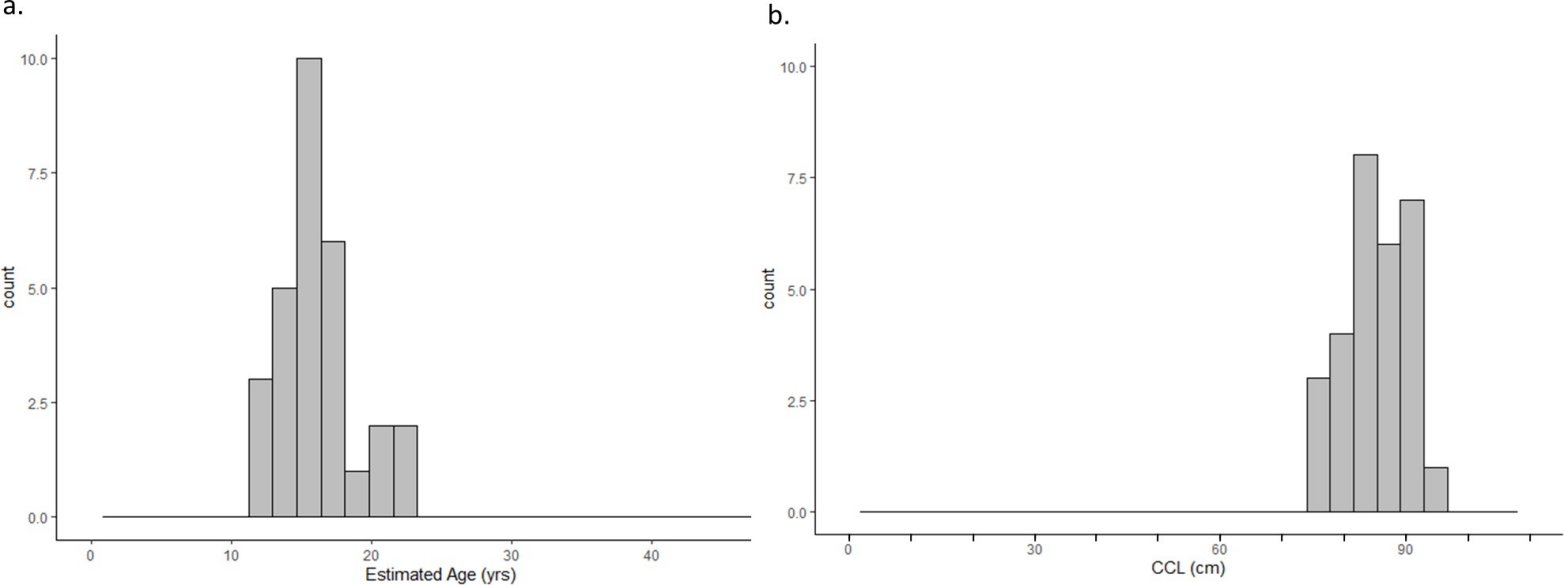
Flatback maturation. Estimated a) age-at-sexual-maturation (ASM) and b) size-at-sexual-maturation (SSM) for n = 29 flatback sea turtles.

We identified 29 turtles with rapprochement growth patterns and that could be aged, indicating the timing of the onset of sexual maturity (Fig 5, S1 Table in S3 File). One bone was excluded from the aging analysis, however, due to it having all retained visible LAGs only in the post-rapprochement section of the bone (resorption core diameter, 32.5 mm), and therefore the onset of maturity could not be detected, and CF1 was deemed not appropriate for this bone. As a result, this sample was excluded from the remaining age analysis and was used in only the size- and growth-based analyses. For the 29 bones we aged, estimates for ASM ranged from 12.0 to 23.0 years (mean ± SE 16.3 ± 0.53, coefficient of variation, CV = 0.18), and SSM ranged from 76.1 to 94.0 cm CCL (mean ± SE 84.9 ± 0.90, CV = 0.06). Estimating CV can be useful to compare the range and variability of ASM and SSM among other sea turtle species and populations (Avens et al. 2017). The number of LAGs observed beyond the onset of maturity (at the rapprochement LAG) represented the observed reproductive longevity [23]and the maximum observed was 31 years (S1 Table in S3 File).

The growth of flatbacks, modeled by both the GAMM smoothing spline and von Bertalanffy curve, showed similar patterns of monotonic growth, slowing as turtles approached maturity at a range of body sizes (Fig 4A and 4B). The mature samples were collected from among three different geographic regions, each showing a range in maturation age and size (Fig 4C). The GAMM smoothing spline, fit to the paired CCL and estimated ages for 72 turtles was significant (p < 0.0001, Edf = 8.832, adj. r^2^ = 0.922), and showed rapid growth through age ~10, slower growth through age ~20, and plateauing around age ~20 as turtles approached maturity above ~80 cm CCL (Fig 4A). The mean SSM, obtained from rapprochement in the current study (84.9 cm CCL), corresponded with a spline-predicted ASM of 18 years (95% CI: 16 to 24 years). When referencing mean nesting sizes reported in the literature (86.4 to 94 cm CCL), the estimated ASM is 24 years and above, as the larger sizes were beyond the model’s maximum (Fig 4A; [27, 65]). Minimum SSM from rapprochement (76.1 cm CCL) yielded a spline-predicted ASM of ~11 years (95% CI: 10 to 12; using skeletochronology, the ASM for this sample was 12 years). When the spline curve was applied to minimum nesting sizes reported in the literature (72 to 85.5 cm CCL from across all the different regions) corresponding minimum ASM was ~9 to ~18 years (Fig 4A; [27, 65, 66]). Larger nesting sizes (>94 cm) were out of the range of the smooth spline and 95% CI, likely due to constraints on sample sizes and representation from the largest adult CCLs.

The bootstrapped von Bertalanffy growth model showed consistencies with the spline curve, increasing steadily through age ~10 and then nearing an asymptote at approximately 89 cm CCL, with minimal growth beyond age 20 (Fig 4B). The model estimated the age-at-sexual-maturation (ASM) for the mean SSM of 85 cm (this study, at rapprochement) at 16.3 ± 0.05 years (95% CI: 12.8 to 27.7 years), the estimated upper size limit, *L*_*inf*_, was 89.2 ± 0.04 cm (95% CI: 85.5 to 95.9 cm) and the intrinsic growth rate parameter, *k*, was 0.185 ± 0.0004 (0.16 to 0.22).

**Fig 6:**
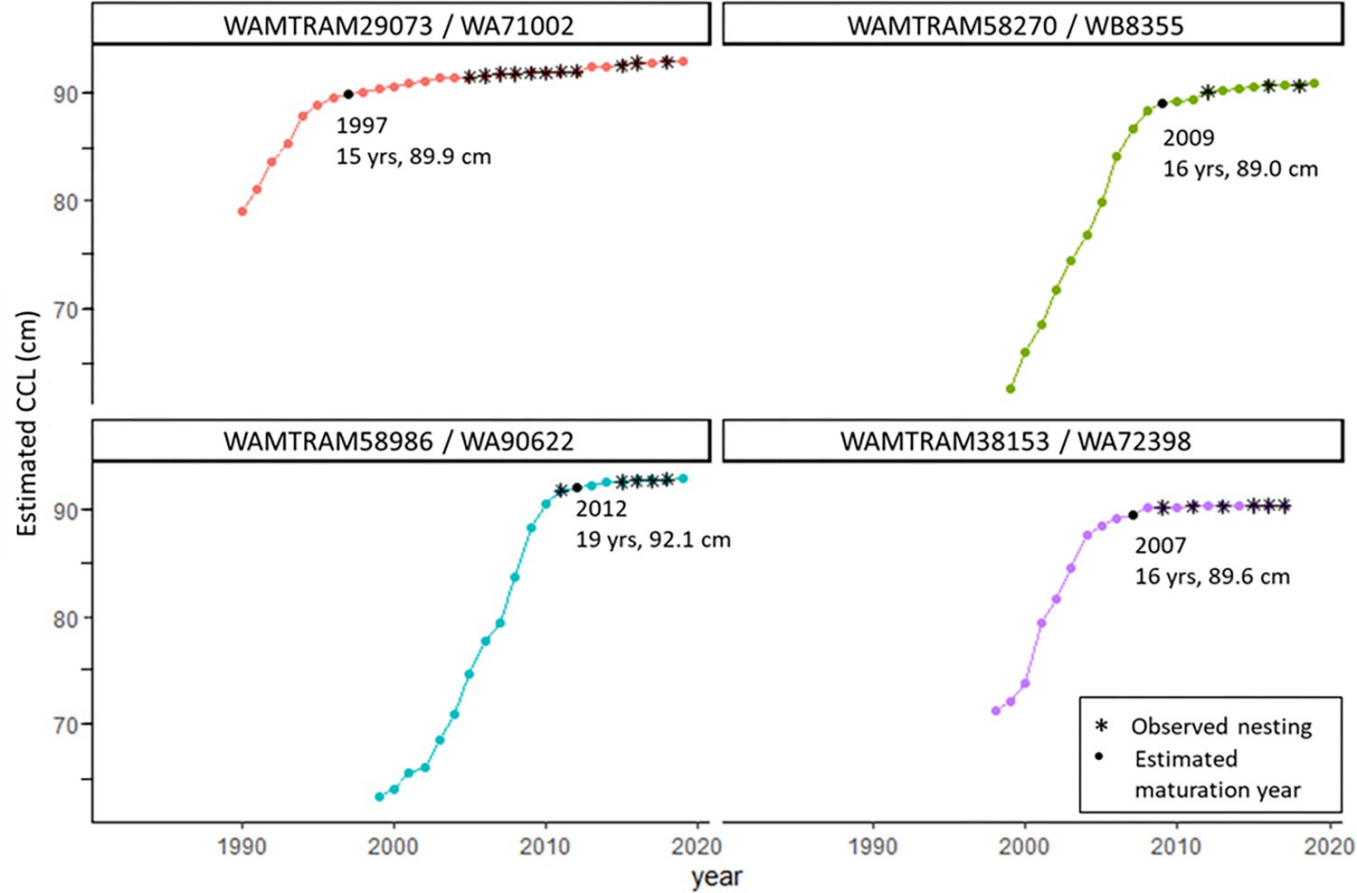
Flatbacks with nesting histories. The skeletochronology-assigned year and curved carapace length (CCL, cm) are shown for four analyzed humeri that were recovered from adult female flatback turtles with previous nesting histories observed during on-going nesting beach monitoring in Western Australia. Filled black circles show the skeletochronology-assigned maturation year for each turtle, with corresponding year, age (years) and CCL (cm) shown. Black asterisks show the year for each observed nesting event for each turtle.

Of the 29 mature adults analyzed, four had previous nesting histories collected during on-going nesting beach monitoring in Western Australia (DBCA unpub; Fig 6). The paired-samples Wilcoxon rank sum test found that the LAG-back-calculated CCL estimates and observed CCL measurements (mean value of all paired samples used for each individual turtle) were not significantly different (p = 0.875), providing support for the use of LAG-based skeletochronology size estimates, as well as supporting the assumption of annual LAG formation in flatback humeri.

## Discussion

### Age and size

These results provide useful baseline data on flatback length-at-age, growth and maturation timing, despite the gap in the size distribution of turtles in the current study. The group of the smaller turtles from which samples were available for analysis were all less than 40 cm CCL in size and less than 4 years old, providing evidence of rapid growth in this early developmental life stage. The group of larger turtles, all greater than 70 cm CCL at death, had a large span in estimated age (15 to 48 years). This is further evidence supporting rapid somatic growth as well as the potential for early maturation of this species, similar to what has been found for some Kemp’s ridleys and hawksbill populations [58, 67]. These findings also help elucidate the drivers behind the uneven size distribution found during in-water monitoring efforts, where most flatbacks observed are either < 40 cm or > 70 cm CCL. For example, during three years of monitoring at Roebuck Bay, out of 130 sea turtles captured, fewer than 10 were in the 50–70 cm CCL size range, with the smallest non-post-hatchling to date being 56.5 cm CCL (DBCA unpub.). While it is not yet known why flatbacks in the 40 to 70 cm range are so rarely encountered, there are a few ideas related to growth rates, habitat use, and predation that could help explain this observation. First, turtles in this size range may experience rapid growth such that there is only a small window of time for them to be observed. Second, it could be driven by habitat use during this juvenile stage, where turtles may occupy more remote and offshore areas and not easily accessible by researchers. Finally, it may be related to the large predators that are common in flatback habitats, and therefore any turtles in this size range that may die for any reason are consumed by predators at sea and therefore are highly unlikely to be washed ashore and recovered as a stranded carcass. Next, the CV for ASM of flatbacks (0.18) was slightly smaller compared to the ASM CV previously reported for Kemp’s ridleys (0.29) and loggerheads (0.22), suggesting that age to maturation may be slightly less variable for flatbacks in comparison to these other sea turtle populations. Yet additional research is recommended given the smaller sample size in the current study, and ideally future studies will have sufficient sample sizes to be able to conduct analysis for each stock individually. For SSM, the CV for flatbacks (0.06) was in between the CV reported for Kemp’s ridleys 0.05 and loggerheads (0.07; [48, 55]). The relatively low variability (CV) for ASM, but relatively mid-range CV for SSM of flatbacks, when compared to other sea turtle populations, is also worth further exploration, as it could indicate potential differences in growth rates that may affect the time it takes for turtles to reach maturation sizes. The wide range in sizes of mature flatback sea turtles (SSM range from skeletochronology: 76.1 to 94.0 cm) and their minimal growth post-sexual-maturation, shows flexibility in the growth potential for flatback sea turtles from all regions in Australia (Figs 4 and 5). Future research is necessary to further elucidate the key mechanisms driving these resulting size- and age-at-sexual-maturation differences, which may be related to environmental conditions, foraging behavior, and / or genetically linked.

### Growth

Flatback hatchlings are known to grow rapidly, similar to other sea turtle species, as documented by various flatback head-start programs and studies using captive-raised hatchlings. Salmon et al. [36] reported that “individual [neonate] turtles varied considerably in growth rates”, starting at mean ± SD 6.66 ± 0.60 cm straight carapace length (SCL), and growing to 9.43 ± 0.34 cm SCL by 7 weeks post-emergence. Similarly, captive-raised flatbacks from Eastern and Western Australia were a mean size (SCL) of 12.7 cm by 11 weeks of age and 12.6 cm by 19 weeks of age, respectively [68].

Sea turtles are known to grow at variable rates due to innate biological (e.g., populations, sex), and environmentally-dependent factors, including water temperatures (e.g., [69–71]), quality and abundance of food resources (e.g., [72]), compensatory growth (e.g., [73, 74]), foraging population density (e.g., [72, 75]), and some of these conditions change as sea turtles grow and shift into different habitats. The absence of an oceanic juvenile stage for flatbacks, which occupy neritic shelf ecosystems during their entire lifetime, eliminates the drastic ontogenetic habitat shift most other hard shelled sea turtle species undergo, and as a result, this could reduce the relative diet variability flatback sea turtles experience as they grow from hatchlings to adults. These same life history traits also contribute to the lack of understanding about neonate and juvenile habitat-use, diet, and growth rates. Future research examining diet and trophic levels of all life stages of flatbacks will help further describe this life history pattern. This can be especially useful for growth modeling given that in juveniles of other sea turtle species, changes in diet and habitat have been shown to influence somatic growth rates (e.g., [72, 76, 77]). Classic growth and ontogenetic shift theory states that a habitat shift is likely to occur for an individual when the new habitat (and prey within) provides an advantage—typically increased growth and or survivorship—over the original habitat [78]. For some sea turtle species, growth has been shown to decrease (e.g., [79, 80]) and in some cases increase (e.g., [77, 81]) prior to a turtle shifting from an oceanic habitat to a neritic habitat, and then increasing again a year or two after the habitat shift has occurred (e.g., loggerheads [77]; Kemp’s ridleys [47, 55]; green sea turtles [49]). The result is that some sea turtle species show evidence of non-monotonic growth during development with a slight growth-increase observed during or after settlement to a neritic foraging ground. Yet the results from the current study show this is likely not the case for flatback sea turtles, as their growth decreased more monotonically while they grew and developed in a more constant neritic habitat throughout their entire lives (S3 Fig in S3 File), further supporting the life history pattern void of an oceanic juvenile stage. Ongoing studies focused on flatback diet and trophic level from hatchling stage to adulthood, at all known foraging regions, are helping to better describe drivers for flatback sea turtle growth rates. Studies employing a variety of techniques, including stomach content analysis, lavage and mouth samples from live captures, satellite tagging of turtles from nesting beaches to foraging grounds, and stable isotope analysis studies, will all help inform the influence of diet on growth and reproduction rates for flatback sea turtles [27, 82, 83] (DES Queensland Turtle Conservation Project, DBCA unpub.).

The estimated growth of the 29 mature turtles in the current study was minimal after sexual maturity and corresponded with published growth rate data for most sea turtle species, including flatbacks. In the current study, mean yearly growth rates for turtles in the 70–80, 80–90, and 90+ cm CCL size classes were 1.36 ± 0.12, 0.86 ± 0.08, and 0.19 ± 0.03 cm, respectively. At Peak Island in the Great Barrier Reef, mean ± SD yearly growth rates of nesters were 0.012 ± 0.21 cm [84], and in the Gulf of Carpentaria were 0.101 ± 0.24 cm [85]. The higher growth rate for the smaller putative-adult size classes likely reflects 1) that some individuals in these size classes are not yet mature and are still experiencing higher somatic growth rates, and 2) that after maturity, there is evidence of continued growth, given that neophyte (primigravid) nesters averaged 1.95 cm smaller in comparison to remigrant nesters, as observed for the east Queensland population [27], indicating that it is common for female sea turtles to grow 1–2 cm after first nesting. Comparisons of the range of body sizes of putative primigravid vs. returning nesters at Bare Sand Island also supports continued growth after first breeding (CCL range and mean: 78–89.1 cm, 84.3 ± 3.0, n = 25 vs. 77.6–92.3 cm, 85.5 ± 2.9, n = 127; M. Guinea pers comm.). Lastly, tagging at foraging grounds and nesting beaches has also provided evidence suggesting that turtles’ growth may slow for 1–10 years prior to first nesting, and so it is possible that ASM based on plateauing growth could be an overestimation (Limpus, unpub.). It has been shown for other sea turtle species to have decreased somatic growth near or after reaching maturation, and variation in size-at-sexual-maturation and associated growth rates has also been well documented [86, 87]. Additional work combining both mark-recapture and skeletochronology will be useful for refining ASM estimates for flatback sea turtles.

The results from the current study also indicate possible differences in growth rates for flatback sea turtles in different regions which experience different environmental regimes (i.e. Great Barrier Reef vs. Gulf of Carpentaria, vs. northwestern shelf) and/or belong to genetically distinct breeding groups [30], and these possible effects on growth should be further explored (Fig 4C). Similarly, the difference observed between one male and one female both recovered in the Northern Territory prompts future research questions exploring possible sexual dimorphism among flatback sea turtles (Fig 4C). Yet in order to fully evaluate any of these potential differences, more information is needed for all the samples analyzed, such as sex, nesting origin, and residencies, and these details were not known for many of the samples used in the current study. Hopefully continued bone collection efforts, together with ongoing mark-recapture studies, will help further address these important research questions. With increased flatback sample sizes from discrete groups, future studies will be able to better refine these age-specific vital rates, such as growth and age- and size-at-sexual-maturation, for individual flatback stocks.

### Sexual maturation

The mean SSM estimated for the 29 mature turtles in the current study, based on skeletochronology rapprochement, is 84.9 ± 0.9 cm CCL, and corresponds with the reported minimum nesting size for both Western Australia and Eastern Australia rookeries (85 cm CCL [65] and 85.5 cm CCL [27], respectively), from where nearly all the mature bones were recovered (Table 2). But this mean SSM from the current study is larger than the minimum nesting size reported in the Northern Territories (72 cm CCL [66]) and for the Torres Strait stock in the Gulf of Carpentaria (80.5 cm CCL [27]; Table 2). When comparing the mean nesting sizes across these four regions, flatbacks in Eastern Australia are typically larger (94 ± 2.6 cm CCL [27, 88]) than those observed nesting at Western Australia rookeries (90 ± 2.5 cm CCL, range 85 to 99 cm CCL, n = 73, 4 rookeries [65]; 88.7 ± 2.6 cm CCL n = 241, Mundabullangana, [89 in 27]) and Gulf of Carpentaria (89.3 ± 2.7 cm CCL [90]), with turtles at Northern Territories having the smallest mean nesting sizes (86.4 ± 0.26 cm CCL n = 257, Field Island [66]; Table 2).

**Table 2:** Flatback maturation size and age.

Region	Source	CCL (cm) Mean ± SD	CCL (cm) Range (min to max)	ASM (yr) Skeleto-estimated	ASM (yr) Spline-predicted
All (mostly WA, EA)	this study	84.9 ± 0.9	76.1 to 94.0	16.3 ± 0.53 (12 to 23)	18 (CI: 16–24)
Eastern Australia	[27,88]	94 ± 2.6	85.5 to 100	-	18+
Torres Strait (Gulf of Carpentaria)	[90]	89.3 ± 2.7	80.5 to 97	-	14+
Northern Territory	[66]	86.4 ± 0.26	72 to 96.5	-	9+
Western Australia	[65]	90 ± 2.5	85 to 99	-	18+

Size-at-sexual-maturation (SSM) and age-at-sexual-maturation (ASM) for flatback sea turtles throughout Australia. Estimates from the current study, obtained through skeletochronology and spline-predicted-ASM are presented to compare to observed mean, minimum and maximum nesting sizes from the literature. Corresponding spline-predicted ASMs are provided for each region based on the minimum nesting CCL reported in the literature from each region.

A closer examination of the distribution of SSM estimated from the bones of turtles collected at these two main regions represented in this study (Western Australia and Eastern Australia) highlights potential differences in body size between flatbacks in these different stocks (Fig 4C). The Western Australia flatback samples (n = 20) had a more narrow range of SSM (mean ± SE 85.8 ± 0.8 cm CCL) in comparison to the Eastern Australia turtle samples (n = 4, mean ± SE 85 ± 4.46 cm CCL) suggesting that the two different breeding stocks may potentially have different distributions in maturation sizes (Fig 4C, S1 Table in S3 File). In contrast to the body size differences, the distribution of ASM estimates between the two groups were more similar in variation, but with the Western Australia samples maturing slightly younger with ASM of mean ± SE 15.7 ± 0.58 years (range: 12 to 22 years), and Eastern Australia samples having ASM of mean ± SE 18.5 ± 0.87 years (range: 17 to 21 years; Fig 4C).

The current study also included three samples collected from mature turtles in the Northern Territory (SSMs: 77.7, 78.0, 89.8 cm CCL), where mean nesting size reported in the literature is at the small end for all flatback sea turtles, and is similar to the mean SSM in the current study (mean ± SE all bones: 84.9 ± 0.9 cm CCL). At the Northern Territory site, Field Island, [66] reported mean ± SD nesting sizes for 257 flatbacks at 86.3 ± 0.26 cm CCL, and six additional studies at additional Northern Territory locations, summarized in [27], reported means between 85.5 and 89.3 cm CCL, all smaller than the means for the Eastern Australia stock. The ASMs for the three mature turtles from the Northern Territory were: 15 years for one male turtle, 18 years for one female turtle, and 23 years for an unknown-sex turtle (S1 Table in S3 File).

Finally, the Torres Strait stock from the Gulf of Carpentaria represented the fourth region covered in the current study, and while it is known to host the largest concentration of flatback nesting, including Crab Island, Kerr Island, and Deliverance Island (reviewed in [27]), there were no bones collected from mature (large) turtles in this region for the current study, so there are no empirical SSM or ASM estimates from this stock. Published literature has shown nesting females in this region average 89 cm CCL [27] (; Crab Island mean ± SD 89.3 ± 2.66 cm CCL, range: 80.5 to 97.0 cm CCL, n = 326 [90]; Crab Island 88.2 ± 3.09 cm CCL, n = 69 [85]; Deliverance Island 88.8 ± 2.96 cm CCL, range: 81.5 to 94.0 cm CCL, n = 18 [91]), with one male at Crab Island recorded at 83.1 cm CCL [90]. The results of the current study estimate ASM for this mean nesting size (89 cm CCL) at 14+ years (Table 2), and future studies applying skeletochronology to bones from this stock are needed to better refine these estimates of maturation timing.

In comparison to other sea turtle species, flatbacks are likely to reach maturity before 20 years of age, which is in the range estimated for Kemp’s ridleys, hawksbills, leatherbacks, and some populations of greens and loggerheads (see review in [9]). Yet the flatbacks tend to mature at a larger size, relative to the other species of hard-shelled sea turtles with more similar ages at maturity, thus suggesting that flatback sea turtles may grow faster in their juvenile life stage. This makes flatbacks somewhat similar to leatherback sea turtles, a species in their own family (*Dermochelyidae*), and one that has been shown to grow rapidly to reach maturity at a large size (mean 129 cm CCL) and a mean age at maturity ranging from 17–19 years [23]. It is interesting to note that both flatback sea turtles and leatherback sea turtles follow a different life history and habitat use pattern than the other sea turtle species. Bolten [32] described three basic life history patterns, the most common being the oceanic-neritic developmental pattern and is characteristic of all the hard-shelled sea turtle species except for the flatback. The flatback pattern was characterized as a neritic developmental pattern, while the leatherback life history pattern was an oceanic development pattern [32]. In addition, the intrinsic growth parameter (*k*) estimated in the current study (0.19) is similar to that of the hard-shelled sea turtles (e.g. Kemp’s ridley, 0.20, reviewed in [10]) that have documented relatively short (≤ 2 years) oceanic developmental stages, suggesting growth could also be related to duration as well as location of developmental patterns. Further exploration into the specific mechanism of these unique life history patterns of both the flatback and leatherback species and the facilitation of rapid growth in the juvenile phase is encouraged and will help elucidate the significance of these novel habitat use strategies.

Lastly, we acknowledge some inherent limitations on using skeletochronology for such demographic estimates for sea turtles in general. As with all skeletochronology studies, there is some minimal error associated with LAG identification and measurement, but numerous skeletochronology studies have shown that the results produced are still extremely informative for the characterization of sea turtle populations’ size, age, and growth rates [9, 42, 44]. Next, skeletal-based maturation estimates assume that rapprochement in the LAGs corresponds with the onset of breeding [9, 48, 64], and this would affect the ASM and SSM estimates if turtles continue growing some (~1–2 cm) after their first nesting, or if turtles show slowing growth in the years immediately preceding their first breeding, as discussed above. Future studies that combine long-term nesting histories with skeletochronology will help to refine these skeletal-based maturation estimates, as was done in the current study (S4 Fig in S3 File). These future studies will also continue to validate the assumptions about annual formation of LAGs, as partially addressed here. The four adult turtles with previous nesting histories that were analyzed in the current study provide support for this annularity assumption for flatbacks given that 1) the CCL-back-calculated measurements were not significantly different from the CCLs observed during nesting events (p = 0.875), and 2) the number and positioning of the years for which each turtle was observed nesting aligned well with the sequence of LAG-indicated mature years (Fig 6).

As a result of homogenizing the samples from the current study, we acknowledge that the findings represent a baseline for age, size, and growth-based vital rates of flatbacks in general, with bias toward the Western Australian stocks, where most of the samples were collected. In the current study, all samples needed to be homogenized into one group despite them being recovered from along the entire northern coast of Australia, due to the limited number of samples that exist. Ideally, bones for a single study would be grouped according to how and or where they stranded, such that the results from the age and size analysis could be directly informative about a distinct population (e.g., regionally-specific bycatch [46, 92]; foraging aggregation [49, 93]; cold-stun event [51]; rookery [48]). Future efforts that eventually collect more flatback samples from distinct habitats and breeding or foraging groups will provide more precise demographic parameters for individual stocks and extend results of the current study.

## Conclusions

The age and growth data presented here help address a major data-gap for flatback sea turtles by helping resource managers better estimate important population growth rates using these new estimates of somatic growth rates and age at maturity. The skeletochronology-derived age and growth data presented here characterize the length-at-age relationship, and establishes baseline growth rates from the hatchling to adult life stages, in addition to producing empirical estimates of age-at- and size-at-sexual-maturation (ASM, SSM) for the flatback species as a whole. The range of estimated ASM is at the lower end among other sea turtle species, suggesting more rapid growth and development for this neritic-dwelling sea turtle. The estimated ages of the mature turtles (12.0 to 23.0 years) and the maximum observed reproductive longevity (31 years) support previous findings obtained through long-term mark-recapture studies. These findings are directly applicable to ongoing population assessments and for informing management and conservation planning throughout northern Australia. The study take-aways are directly applicable in conservation planning because previously unknown demographic parameters for flatback sea turtles (ASM, SSM, growth rates) are fundamental updates in stock viability models to better assess management approaches such as stock-specific critical habitat designations.

## Supporting information

S1 File Flatback LAG data. Basic information provided for each flatback bone processed for skeletochronology.
(XLSX)

S2 File Flatback demographic data. The demographic data provided for each flatback bone analyzed (age, CCL and growth).
(XLSX)

S3 File Additional info. Contains supporting tables and figures.
(PDF)
