# Supplementary material for: Age-specific growth and maturity estimates for the flatback sea turtle (Natator depressus) by skeletochronology
Source: PLoS One. 2022 Jul 20;17(7):e0271048. doi: 10.1371/journal.pone.0271048 (PMC9299290; doi:10.1371/journal.pone.0271048)

Supporting information

Tables

S1 Table - Summary information on the mature turtles (n=29) showing stranding location, sex (F = female, M = male, U = unknown), final estimated age, final CCL (cm), age-at-sexual-maturation (ASM, years), size-at-sexual-maturation (SSM, cm), and reproductive longevity (years).

ID	Location	Sex	Age (final, yrs)	CCL (final, cm)	ASM (yrs)	SSM (cm)	Reproductive longevity (yrs)
1	Eastern Australia	U	43	79.5	18	76.1	25
2	Eastern Australia	U	27	83.9	18	78.7	9
3	Eastern Australia	U	48	93.4	21	91.2	27
4	Eastern Australia	U	36	96	17	94	19
5	Northern Territory	U	29	78.8	23	78	6
6	Northern Territory	M	33	80.5	15	77.7	18
7	Northern Territory	F	22	91.2	18	89.8	4
8	Western Australia	U	17	82.2	12	77.6	5
9	Western Australia	U	22	83	16	81.5	6
10	Western Australia	U	18	85	14	82.4	4
11	Western Australia	F	19	85	12	82.3	7
12	Western Australia	F	17	85	14	82.9	3
13	Western Australia	F	31	85.5	18	83.5	13
14	Western Australia	U	20	86	18	84.2	2
15	Western Australia	F	27	86.4	15	84.5	12
16	Western Australia	F	18	87.3	15	85.7	3
17	Western Australia	F	16	87.4	14	85.8	2
18	Western Australia	F	27	88	22	87.2	5
19	Western Australia	M	45	89.5	14	84.5	31
20	Western Australia	F	29	89.6	13	86.6	16
21	Western Australia	U	42	90.4	15	87.4	27
22	Western Australia	F	26	91	16	89.6	10
23	Western Australia	U	27	91.1	20	89.5	7
24	Western Australia	U	21	91.4	16	89.5	5
25	Western Australia	F	26	91.8	16	89	10
26	Western Australia	F	26	92.9	19	92.1	7
27	Western Australia	F	37	92.9	15	89.9	22
28	NA	F	23	80.5	16	79.7	7
29	NA	F	17	83	12	82.2	5

S2 Table - Growth rates derived from back-calculated incremental growth layers and aligned with estimated age (years) and size (CCL, cm) which have been grouped into a) age classes and b) size classes.

a.

Age class (yr)	mean growth	sd	n	SE
[1-3)	12.4	5.80	19	1.33
[3-5)	6.8	3.8	2	2.65
[5-10)	2.7	1.91	63	0.24
[10-15)	1.9	1.38	138	0.12
[15-20)	0.88	1.1	138	0.10
[20-25)	0.21	0.22	94	0.02
[25-30)	0.16	0.23	68	0.03
[30-35)	0.11	0.09	36	0.01
[35-40)	0.06	0.05	25	0.01
[40-45)	0.08	0.07	17	0.02
[45-50)	0.03	0.01	5	0.004

b.

Size class (cm)	mean growth	sd	n	SE
(10,20]	11.70	1.29	7	0.49
(20,30]	12.90	7.29	12	2.11
(30,40]	6.45	3.23	2	2.29
(50,60]	1.72	1.07	8	0.38
(60,70]	2.58	1.79	47	0.26
(70,80]	1.36	1.57	163	0.12
(80,90]	0.86	1.20	256	0.08
(90,100]	0.19	0.29	134	0.03

Figures

S1 Figure. Relationship between turtle body size (curved carapace length, CCL, cm) and total humerus section diameter (THD, mm) for the 59 turtles with measured CCL (*black*) and the 14 turtles that were missing CCL measurements and had CCL estimated based on CCL:THD relationship (*red*).

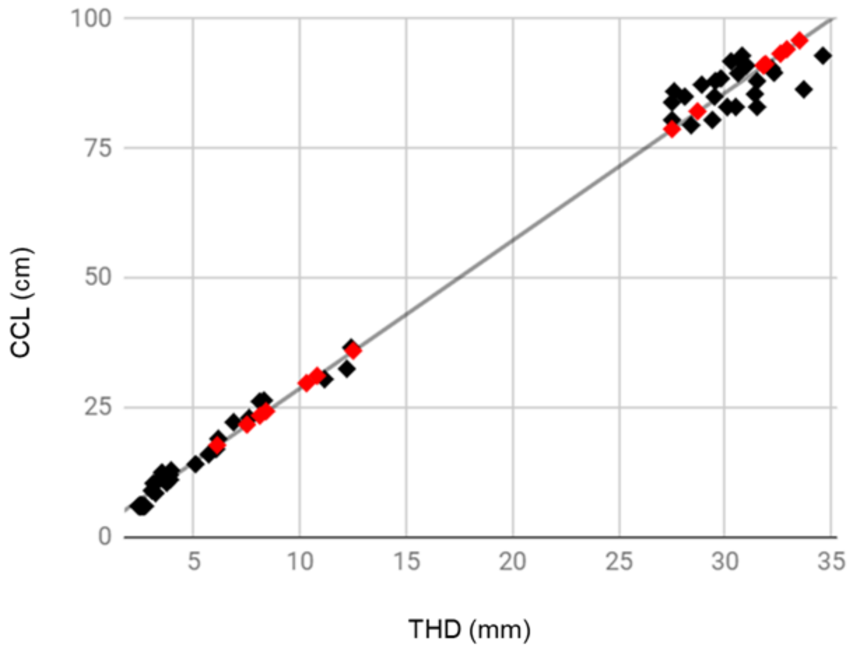

S2 Figure. Relationship between LAG diameter and LAG number from the bones which retained an annulus (Group 1, n=15 turtles, 23 LAGs) used to generate the best-fit correction factor equation (*see Methods and Results*).

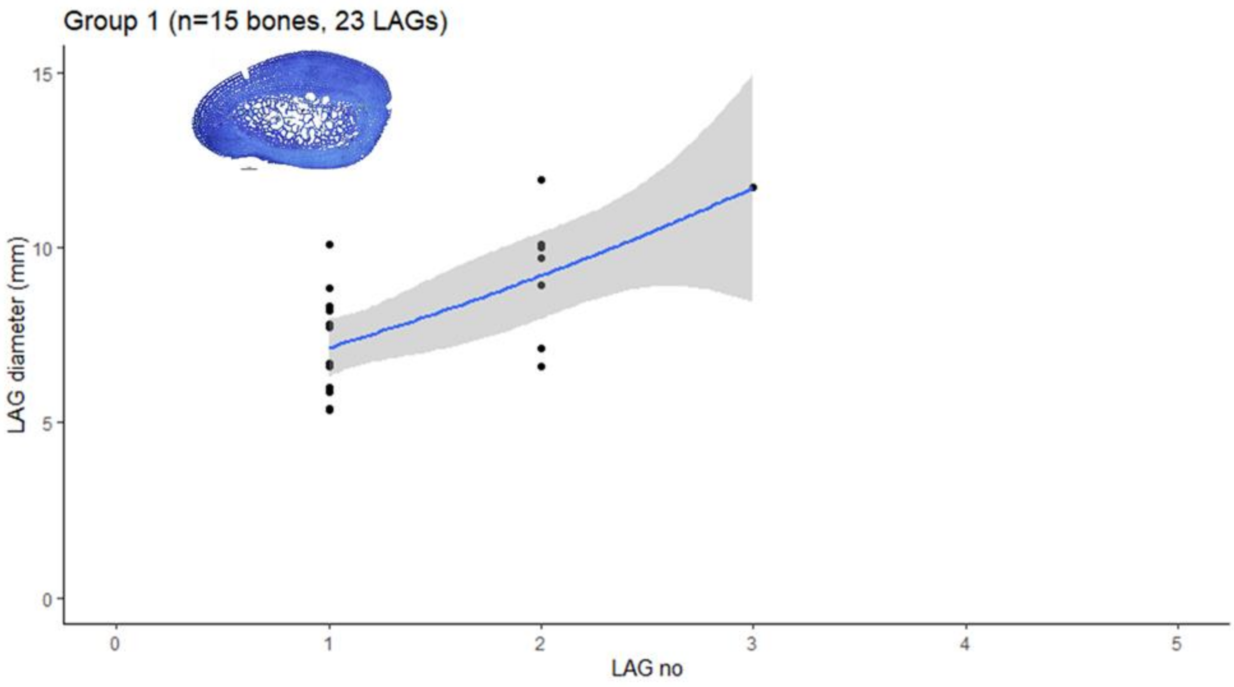

S3 Figure. Annual growth by a) age class, and b) size class.

a.

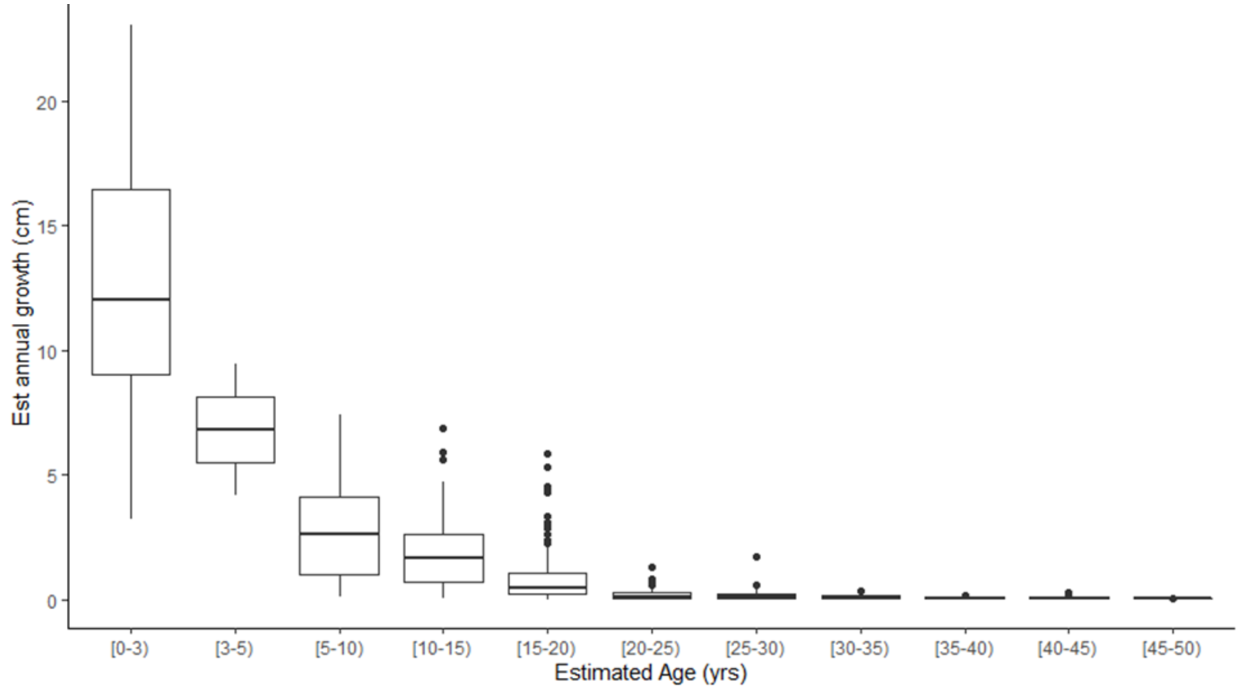

b.

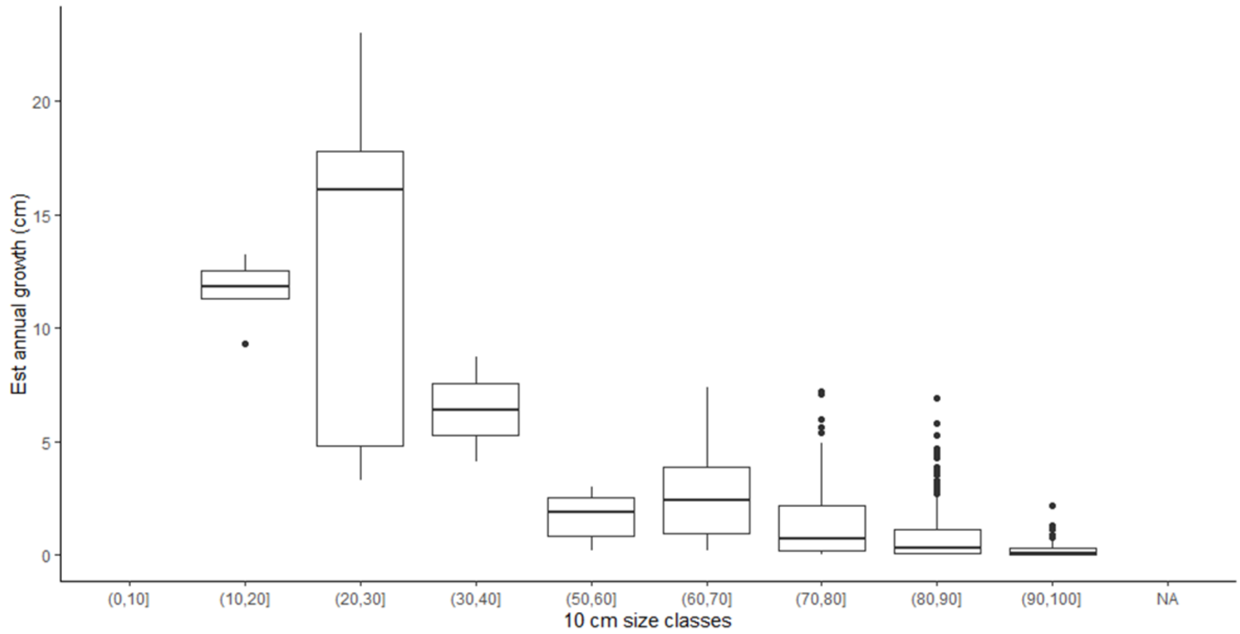

S4 Figure. Paired CCL values for the 4 turtles with nesting histories. Back-calculated CCL estimates on y-axis, and measured CCL values on the x-axis, color coded for each turtle. The dashed black line shows the 1:1 relationship if all paired values were exactly the same.

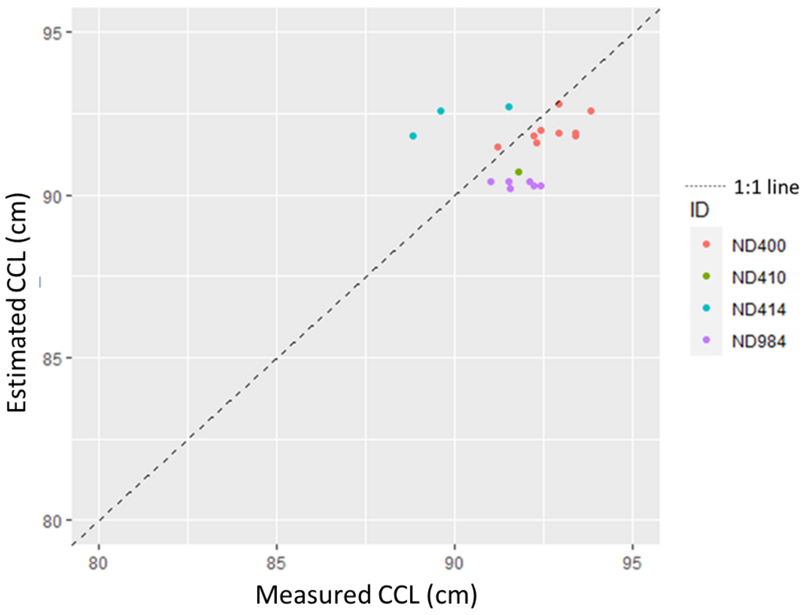

Supplement: S3 File — Contains supporting tables and figures. (PDF) [file pone.0271048.s003.pdf]
